# Basolateral Sorting of Syntaxin 4 Is Dependent on Its N-terminal Domain and the AP1B Clathrin Adaptor, and Required for the Epithelial Cell Polarity

**DOI:** 10.1371/journal.pone.0021181

**Published:** 2011-06-15

**Authors:** Elena Reales, Nikunj Sharma, Seng Hui Low, Heike Fölsch, Thomas Weimbs

**Affiliations:** 1 Department of Molecular, Cellular, and Developmental Biology and Neuroscience Research Institute, University of California Santa Barbara, Santa Barbara, California, United States of America; 2 Department of Cell and Molecular Biology, Northwestern University, Chicago, Illinois, United States of America; Institut Curie, France

## Abstract

Generation of epithelial cell polarity requires mechanisms to sort plasma membrane proteins to the apical and basolateral domains. Sorting involves incorporation into specific vesicular carriers and subsequent fusion to the correct target membranes mediated by specific SNARE proteins. In polarized epithelial cells, the SNARE protein syntaxin 4 localizes exclusively to the basolateral plasma membrane and plays an important role in basolateral trafficking pathways. However, the mechanism of basolateral targeting of syntaxin 4 itself has remained poorly understood. Here we show that newly synthesized syntaxin 4 is directly targeted to the basolateral plasma membrane in polarized Madin-Darby canine kidney (MDCK) cells. Basolateral targeting depends on a signal that is centered around residues 24–29 in the N-terminal domain of syntaxin 4. Furthermore, basolateral targeting of syntaxin 4 is dependent on the epithelial cell-specific clathrin adaptor AP1B. Disruption of the basolateral targeting signal of syntaxin 4 leads to non-polarized delivery to both the apical and basolateral surface, as well as partial intercellular retention in the trans-Golgi network. Importantly, disruption of the basolateral targeting signal of syntaxin 4 leads to the inability of MDCK cells to establish a polarized morphology which suggests that restriction of syntaxin 4 to the basolateral domain is required for epithelial cell polarity.

## Introduction

Epithelial cells constitute a large proportion of cells in most major body organs such as skin, liver, kidney and gut [1], [2]. The function of epithelial cells is dependent on the polarized distribution of plasma membrane proteins into apical and basolateral domains [3]. Establishment and maintenance of cell polarity depend upon the precise targeting of apical and basolateral cargo to the respective membranes [3], [4]. A large number of proteins have been identified which mediate and regulate polarized membrane traffic including SNARE proteins [5] which catalyze membrane fusion. Membrane fusion is mediated by the formation a specific complexes between cognate SNAREs on the vesicles and target membranes, which contributes to the specificity of trafficking in all eukaryotes [6]. These proteins have been implicated in the determination of rate and specificity of several fusion steps in polarized pathways [3], [7].

Epithelial cells contain at least two different plasma membrane t-SNAREs, syntaxin 3 and syntaxin 4, exclusively localized to the apical and basolateral membrane, respectively, in a wide variety of epithelial cell types investigated to date [8], [9]. Even before the establishment of proper cell polarity syntaxin 3 and syntaxin 4 localize to sub-micron size separate clusters on the plasma membrane [10]. Studying apical sorting of syntaxin 3, we have previously shown that the correct polarized localization of syntaxin 3 at the apical membrane is essential for the overall maintenance of epithelial polarity [11]. The high degree of conservation of the basolateral polarity of syntaxin 4 suggests that syntaxin 4 function and proper localization may play an equally important role in epithelial polarization.

Basolateral sorting signals are commonly located in cytoplasmically exposed regions and include tyrosine motifs, dileucine and monoleucine motifs and some other non-canonical motifs [12]. Some of these motifs can be recognized by clathrin adaptors which are involved in the identification of cargo and in the formation of clathrin coated vesicles [4], [13]. To date, four major heterotretameric clathrin adaptor complexes have been identified in mammals, AP1-4, two of which have been implicated in basolateral sorting, the AP1 variant AP-1B and AP4 [14]. AP1 is composed by four subunits; γ1, β1, μ1, σ1 and the two closely related AP-1 complexes, AP1A and AP1B, differ only in the incorporation of the respective sorting-signal binding subunits μ1A and μ1B [15]. AP1B is mainly expressed in polarized epithelial cells such as Madin-Darby canine kidney (MDCK) cells [15], where it participates in recycling as well as in the biosynthetic route to the basolateral plasma membrane from recycling endosomes [16], [17].

Fusion of AP-1B vesicles at the basolateral membrane depends on the SNARE protein cellubrevin, which is incorporated into AP-1B vesicles and on syntaxin 4 at the target membrane [18]. These data indicate that syntaxin 4 plays a critical role at the basolateral membrane, yet how syntaxin 4 is selectively incorporated into the basolateral membrane has remained unknown. In this study, we demonstrate that the N-terminal domain of syntaxin 4 is critical for its basolateral localization, and that targeting depends on AP1B. Mutation of this targeting signal leads to non-polarized plasma membrane location and partial intracellular retention of syntaxin 4 in the trans-Golgi network. Furthermore, expression of mis-targeted syntaxin 4 inhibits the ability of epithelial cells to correctly polarize suggesting that the restriction of syntaxin 4 to the basolateral plasma membrane domain is a requirement for the establishment of epithelial polarity.

## Results

### Newly synthesized syntaxin 4 is directly targeted to the basolateral surface

**Figure 1:**
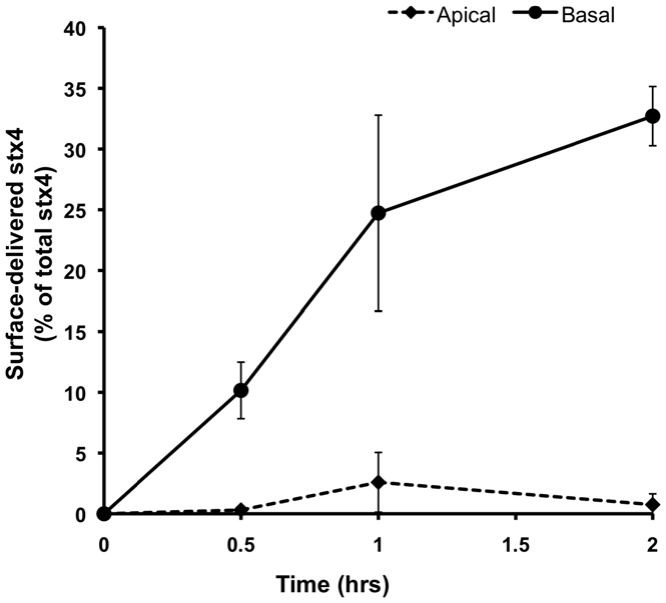
Syntaxin 4 is directly transported to the basolateral surface in MDCK cells. Polarized MDCK cells stably expressing myc-tagged syntaxin 4 were metabolically labeled for 15 min with [^35^S] methionine, followed by a chase for the indicated periods of time. Anti-myc antibody was present throughout the chase in the apical or basolateral media compartment. After cell lysis, surface-delivered, antibody-captured syntaxin 4 was recovered by precipitation with Protein A, and the remaining intracellular syntaxin 4 was captured by immunoprecipitation. Quantification was done by SDS-PAGE and radio-analysis, and the surface-delivered syntaxin 4 was calculated as a percentage of total radiolabeled syntaxin 4.

At steady-state, syntaxins 3 and 4 are localized almost exclusively to the apical and basolateral surface, respectively, of MDCK cells and several other epithelial cells [8]. We have previously shown that a significant fraction of newly synthesized syntaxin 3 is initially targeted to the “incorrect” basolateral plasma membrane domain [11] and must consequently be sorted at a later point by an unknown mechanism. To test whether newly synthesized syntaxin 4 is delivered exclusively to the basolateral membrane or directed to both membranes, apical and basolateral, we used a pulse-chase assay combined with surface immunoprecipitation similar to the method previously used to investigate syntaxin 3 [11]. Because syntaxin 4 lacks an extracytoplasmic domain, we used MDCK cells stably expressing syntaxin 4 containing two C-terminal myc epitope tags. These epitope tags are accessible to anti-myc antibody added to the culture medium of intact cells and do not interfere with the correct targeting of syntaxin 4 as shown previously [10], [19]. Polarized MDCK cells were labeled with [^35^S] methionine and chased for up to two hours. Subsequently, the fraction of syntaxin 4 delivered to the apical or basolateral surface, respectively, was captured by surface immunoprecipitation. As shown in Fig. 1, the vast majority of newly synthesized syntaxin 4 is captured only from the basolateral surface at all time points. This result indicates that syntaxin 4 – in contrast to syntaxin 3 – reaches its final basolateral plasma membrane destination without prior delivery to the apical membrane.

### A necessary basolateral targeting signal is centered around residues 24–29 of syntaxin 4

**Figure 2:**
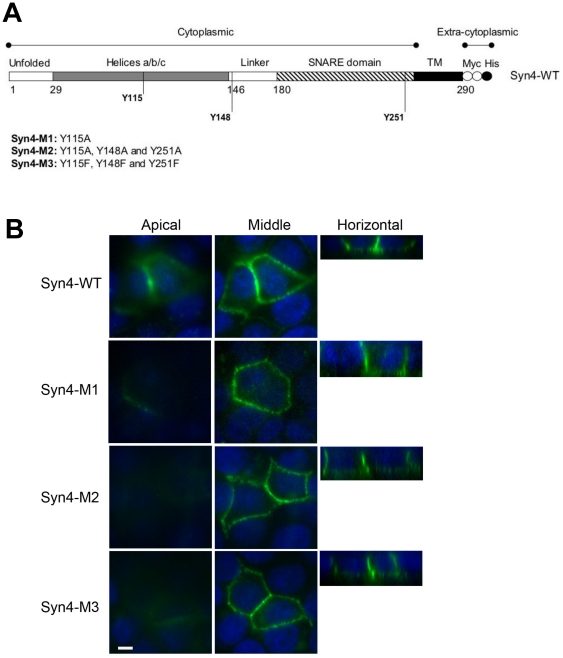
Tyrosine residues are not involved in basolateral targeting of syntaxin 4. (**A**) Schematic representation of mutant syntaxin 4 constructs used. Two myc epitope tags (white circles) and one His_6_ tag (black circles) were added to the COOH termini. Mutants (M1–M3) containing exchanges of Y to A or F are indicated. (**B**) Syntaxin 4 tyrosine mutant proteins transiently expressed in polarized MDCK cells were detected by surface-immunostaining and confocal microscopy. Syntaxin 4, green; nuclei, blue. Representative XY optical sections of the apical region of the cells (left), or the middle of the cells (middle) are shown together with XZ optical section (right). Bar is 5 µm.

To further investigate the mechanism of basolateral targeting of syntaxin 4 we set out to identify the region or motif of syntaxin 4 that is required for basolateral targeting. Several known basolateral targeting motifs contain critical tyrosine residues [17]. The sequence of human syntaxin 4 contains three tyrosine residues, Y115, Y148 and Y251, which are conserved among mammals. To test whether syntaxin 4 contains a necessary tyrosine-based basolateral sorting signal we generated single and combined mutations of these tyrosines to alanines and/or phenylalanines (Fig. 2A). As shown in Fig. 2B, none of these tyrosine mutants alter the basolateral-specific location of syntaxin 4 in MDCK cells suggesting that syntaxin 4 does not contain a tyrosine-based basolateral sorting signal.

**Figure 3:**
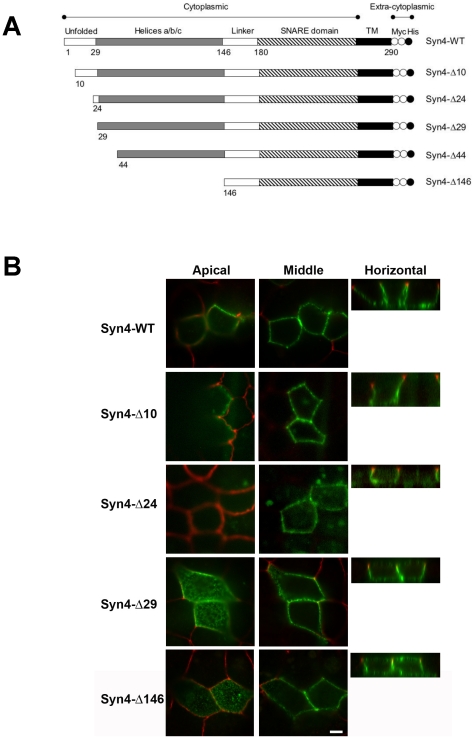
The N-terminal 29-residue domain of syntaxin 4 is necessary for basolateral targeting. (**A**) Schematics of syntaxin 4 deletion mutant constructs. Two myc epitope tags (white circles) and one His_6_ tag (black circles) were added to the COOH termini. (**B**) Syntaxin-4 deletion mutant proteins transiently expressed in polarized MDCK cells were detected by surface-immunostaining and confocal microscopy. Syntaxin 4, green; tight junction protein ZO1, red. Representative XY optical sections of the apical region of the cells (left), or the middle of the cells (middle) are shown together with XZ optical section (right). Bars are 5 µm.

To identify the regions of syntaxin 4 that are necessary for basolateral targeting, we generated mutants with successively deleted domains (Fig. 3A). These deletion mutants were transfected into MDCK cells and their surface localization was analyzed by confocal microscopy. Deletion in the first 29 N-terminal amino acids, and any further deletion, resulted in a non-polarized surface localization of syntaxin 4 (Fig. 3B). To locate a more specific signal, we generated additional deletion mutants. Deletion of the first 10 or 24 amino acids did not result in the mislocalization of syntaxin 4 compared to wild-type protein (Fig. 3C), indicating that the region between residues 24–29 (ALVVHP) is critical for the basolateral sorting of syntaxin 4.

### AP1B adaptor complex is required for basolateral sorting of syntaxin 4

**Figure 4:**
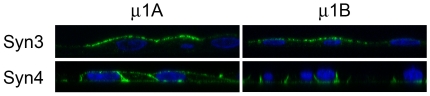
AP1B is required for basolateral sorting of syntaxin 4. (**A**) LLC-PK1:μ1A or LLC-PK1:μ1B cells stably expressing myc-tagged Syn3/Syn4 were grown until confluent and processed for immunofluorescence. Fluorescence staining of syntaxin proteins was performed using anti-myc antibody (1∶400) and DAPI for nuclei staining and analyzed by confocal microscopy.

The epithelial cell specific adaptor complex AP1B is involved in basolateral trafficking of several membrane proteins [18]. To investigate if AP1B is required for the basolateral sorting of syntaxin 4, we took advantage of the renal epithelial LLC-PK1 cell line, which has previously been shown to lack expression of the μ1B subunit and consequently mis-sorts several basolateral proteins, including the transferrin receptor and LDL receptor [20]. We used previously described LLC-PK1 cell lines that were stably transfected to express either the μ1A or μ1B subunits [20]. Transfection with μ1B – but not μ1A - has previously been shown to restore the basolateral sorting defect in these cells [20]. These two cell lines were stably transfected with either myc-tagged syntaxin 4 or syntaxin 3 expression vectors. Surface immunofluorescence showed that syntaxin 4 is localized in a non-polarized manner to both apical and basolateral membranes in the cells expressing μ1A (lacking μ1B) but localizes correctly to the basolateral membrane in the cells that express μ1B (Fig. 4). The apical localization of syntaxin 3 remains unaffected by the presence or absence of μ1B (Fig. 4). These results indicate that the AP1B adaptor is required for basolateral sorting of syntaxin 4 in polarized cells either during biosynthetic delivery, endocytic recycling or both.

### Syntaxin 4-Δ29 is partially intracellularly retained and loses its ability to bind to Munc18c

**Figure 5:**
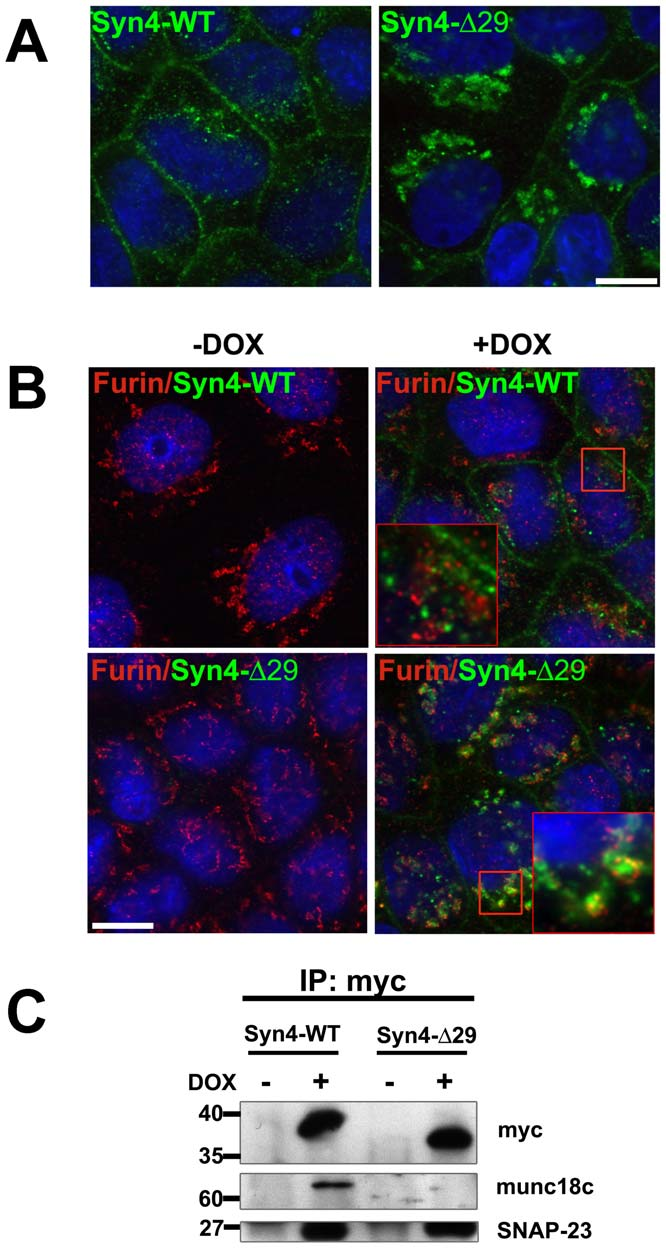
Intracellular localization of syntaxin 4-Δ29 in MDCK cells. MDCK cells stably expressing myc-tagged Syn4-WT or Syn4-Δ29 were cultured to confluence confluent (3–4 days), followed by induction with DOX for 10 hrs. (**A**) Confocal microscopy analysis of immunostained Syn4 after cell permeabilization, green; nuclei (DAPI), blue. (**B**) To further analyze the intracellular location of Syn4-WT or Syn4-Δ29, polarized cultures of MDCK cell lines stably expressing the indicated syntaxin proteins were induced with DOX for 10 hr and visualized by co-staining with monoclonal anti-myc antibody (1∶400) and polyclonal anti-Furin antibody (1∶200). (**C**) Interaction of Syn4-WT and Syn4-Δ29 proteins with SNAP-23 and Munc18c. MDCK stable cells were induced for syntaxin 4 protein expression and immunoprecipitated using anti-myc antibody. Binding of endogenous SNAP-23 or Munc18c was detected by immunoblotting using polyclonal anti-SNAP-23 (1∶3000) and anti-Munc18c (1∶1000).

Sorting of many newly synthesized plasma membrane proteins typically occurs in the *trans*-Golgi network (TGN), where apical and basolateral proteins are selectively packaged into specific transport vesicles for apical or basolateral delivery [21]. Alternatively, cargo may move from the TGN into recycling endosomes where AP-1B can bind its cargo for basolateral delivery [17]. We sought to test whether syntaxin 4 sorting may involve the TGN or post-TGN compartments. To this end, we investigated the intracellular fate of the minimal deletion mutant that caused loss of basolateral-specific surface targeting (syntaxin 4-Δ29, Fig. 3). We generated stably transfected MDCK cell lines expressing myc-tagged syntaxin 4-Δ29 under the control of a doxycycline (DOX)-inducible promoter. In control cells, the majority of wild-type syntaxin 4 is localized to the plasma membrane at steady-state (Fig. 5A). In contrast, a large fraction of syntaxin 4-Δ29 accumulates in large perinuclear structures (Fig. 5A). To characterize this intracellular compartment we performed double immuno-staining with markers of Golgi-associated compartments. Significant co-localization was evident between syntaxin 4-Δ29 and furin, a protein that is primarily localized in the *trans*-Golgi network [22], [23] (Fig. 5B). These results suggest that the export of syntaxin 4 lacking the N-terminal basolateral targeting signal may be stalled in the TGN.

We next tested whether syntaxin 4-Δ29 can still interact with its known binding partners SNAP-23 and Munc18c. Syntaxin 4 binds to the SNARE protein SNAP-23 to form a functional t-SNARE complex [24] and interacts with the SM (Sec1/Munc18-like) protein Munc18c, a regulator of SNARE function [25], [26]. As shown in Figure 5C, deletion of the basolateral sorting signal of syntaxin 4 does not disrupt binding to SNAP-23, but does disrupt binding to Munc18c. The loss of interaction with Munc18c is consistent with previous results [26], [27]. These results suggest that the interaction between syntaxin 4 and Munc18c may be involved in the exit of syntaxin 4 from the TGN and subsequent basolateral targeting.

### Mislocalization of syntaxin 4 inhibits epithelial cell polarity

**Figure 6:**
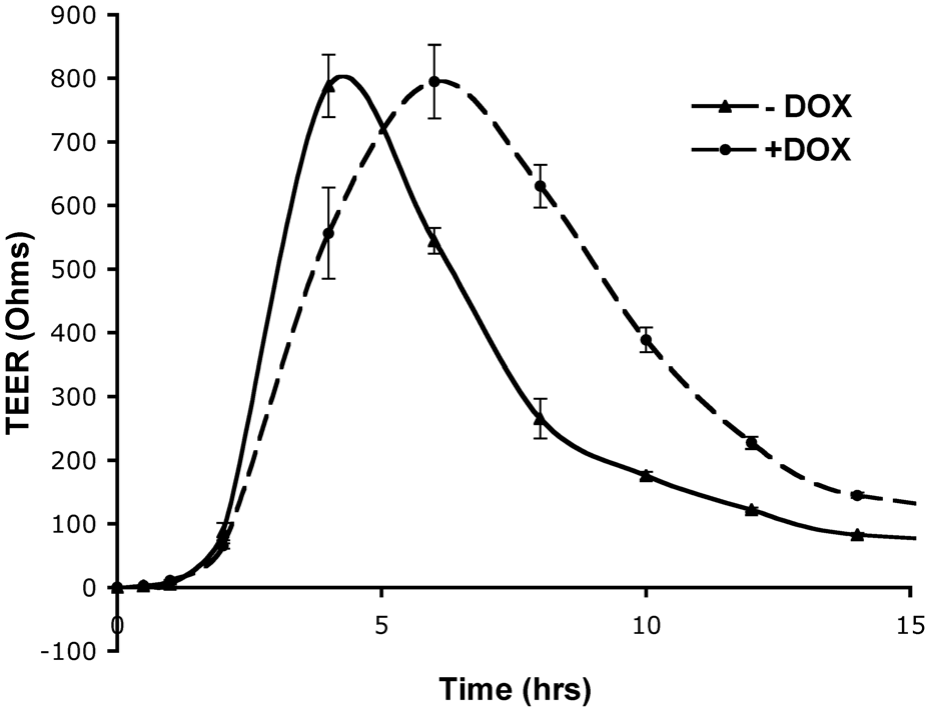
Expression of mistargeted syntaxin 4 causes kinetic delay in tight junction formation. MDCK stable transfected cells for Syn4-Δ29 were cultured on Transwell filters for 72 h, followed by induction with DOX for 8 h. Cultures were switched to low-calcium media for 15 h, resulting in the loss of tight junctions. Cultures were then switched back to normal calcium and the reestablishment of tight junctions was monitored by measuring the TEER (Ohms). Data represent mean values from at least three independent experiments. Error bars indicate SEM.

We have previously shown that the inability to restrict syntaxin 3 localization to the apical plasma membrane domain perturbs the ability of MDCK cells to establish tight junctions and can result in the over-all loss of epithelial polarity [11]. We next tested whether expression of the mis-targeted mutant syntaxin 4-Δ29 may have similar consequences on the ability of cells to polarize. To investigate the kinetics of the formation of the tight junctions, MDCK cells stably expressing syntaxin 4-Δ29 were cultured on permeable filters for four days in the absence of DOX to allow the cells to establish a polarized monolayer. Syntaxin 4-Δ29 expression was induced with DOX for 8 h and cells were subjected to calcium-deficient medium for 15 h, which results in the complete disassembly of tight junctions and consequent loss of trans-epithelial electrical resistance (TEER) [11]. Re-addition of normal calcium leads to the re-establishment of tight junctions and a characteristic peak in the TEER. As shown in Fig. 6, expression of syntaxin 4-Δ29 results in a kinetic delay in the TEER peak by approximately three hours suggesting perturbation of the formation of new tight junctions.

**Figure 7:**
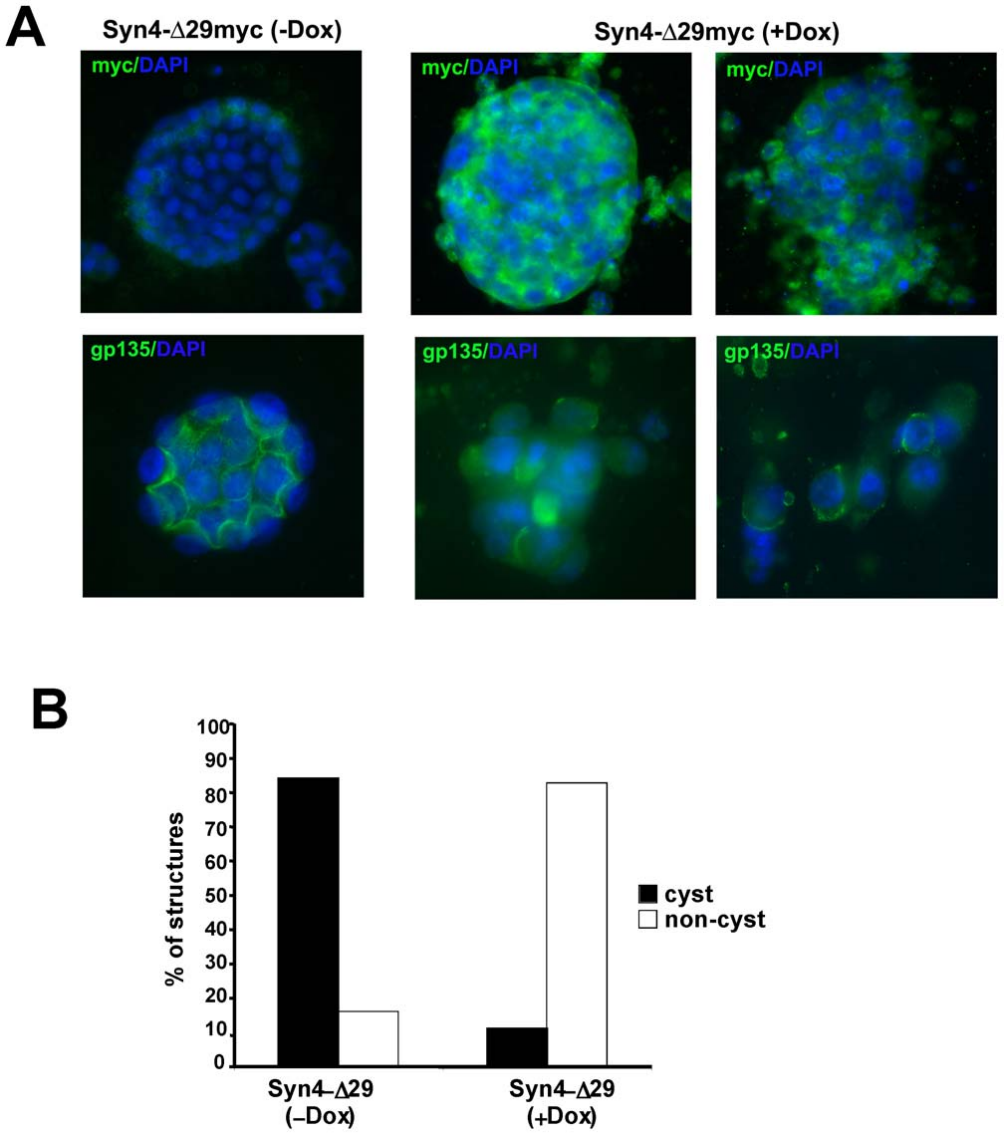
Expression of mistargeted syntaxin 4 prevents cyst-formation in 3D MDCK culture. (**A**) MDCK cells stably expressing Syn4-Δ29 were cultured in 3D collagen. Syntaxin expression was induced with DOX two days after seeding, and culture was continued for an additional 6 days. Cells were fixed and immunostained for GP135 protein, an apical marker, Syn4-Δ29 (myc) and nuclei. Two different panels are shown for cells that express Syn4-Δ29 after induction to represent the variety of defective cysts found in the cultures. (**B**) Quantitation of cyst formation. Cysts consisting of polarized cells or disorganized “non-cysts” consisting of nonpolarized cells (as shown in A) were counted and are expressed as percentage of total structures.

The ability of MDCK cells to establish a polarized phenotype is known to be more sensitive to disruptions of polarity proteins when cells are cultured in 3-dimentional collagen gels as compared to 2-dimentional cultures [1], [28]. We asked whether expression of mis-targeted syntaxin 4-Δ29 may interfere with the development of polarized cells in cysts in 3D culture. Stably transfected syntaxin 4-Δ29 MDCK cells were cultured in collagen gels for 7–9 days in the absence or presence of DOX. Uninduced control cells developed mainly lumen-containing cysts consisting of well-polarized cells. In contrast, syntaxin 4-Δ29 expressing cells largely failed to form cysts but formed disorganized, tumor-like, solid structures lacking lumens and consisting of non-polarized cells (Fig. 7A and 7B). This result suggests that basolateral-specific targeting of syntaxin 4 is required for the establishment of epithelial polarity.

## Discussion

Syntaxin 4 is a widely – if not ubiquitously - expressed SNARE protein that is restricted to the basolateral domain of most epithelial cells studied to date [8]. We have investigated the mechanism of basolateral sorting of syntaxin 4 and have identified a cytoplasmic basolateral sorting signal that is contained in the first N-terminal 29 amino acid residues of syntaxin 4. In addition, we describe a novel role for the AP1B adaptor in syntaxin 4 basolateral sorting. Finally, we show that exclusive basolateral localization of syntaxin 4 is essential for proper epithelial polarization.

Our results indicate that newly synthesized syntaxin 4 is exclusively delivered to the basolateral membrane. This is in contrast to syntaxin 3 which is initially randomly targeted and later sorted to the apical domain [11]. This specific polarized sorting of syntaxin 4 suggests that only one primary route exists for the surface delivery of syntaxin 4 in polarized MDCK cells.

Known basolateral sorting signals include tyrosine-based, di-leucine-, leucine-based and others [12], and these signals are thought to be recognized by specific cytosolic sorting adaptors which mediate protein sorting at specific organelles. We found that basolateral sorting of syntaxin 4 is not dependent on tyrosine motifs. Instead, basolateral sorting depends on the first 29 N-terminal amino acids of syntaxin 4, specifically residues 24–29 (ALVVHP). These results are consistent with a recent study published while this manuscript was in preparation [29]. By mutational analysis these authors identified residues L25 and V26 as essential for basolateral localization of syntaxin 4. Together, both studies clearly identify this region of syntaxin 4 as a necessary basolateral sorting signal.

Torres et al. suggested that the L25/V26 residues resemble a dileucine motif such as those that are required for endocytosis or basolateral targeting in other proteins [29]. Dileucine motifs are known to bind to AP1 adapters [30] which would be consistent with our finding that AP1B is required for basolateral targeting of syntaxin 4.

The N-terminal regions of syntaxins have been described to bind to SM (Sec1/Munc18) proteins [31], which are SNARE regulators involved in vesicle fusion [32]. SM/SNARE complexes can have different binding modes. Originally SM proteins were assumed to bind to closed conformations of syntaxins in which SNARE complex assembly is prevented. However, more recent studies have shown that Munc18a also binds to SNARE complexes [33] as well as monomeric syntaxin 1A [34] and that in these cases formation of SNARE complexes is allowed. SM and SNARE protein interactions are specific, such that Munc18a and Munc18b only bind to syntaxins 1 and 3 whereas Munc18c only binds to syntaxins 2 and 4 [35]. The N-terminal 29 residues of syntaxin 4 have been shown to be required for binding to Munc18c and the three-dimensional structure of the complex has been elucidated [26], [36], [37]. Since we found that efficient surface delivery and basolateral sorting of syntaxin 4 depend on its N-terminal region, and our results confirm that syntaxin 4-Δ29 is not able to bind to Munc18c, this may suggest that formation of the syntaxin 4/Munc18c complex is necessary for surface delivery and/or basolateral sorting of syntaxin 4.

Interestingly, our results show that deletion of the N-terminal domain of syntaxin 4 not only results in non-polarized surface delivery but also that a large fraction of the mutant protein accumulates intracellularly, presumably in the TGN. This suggests that sorting of newly synthesized syntaxin 4 into basolateral transport carriers occurs in the TGN and that syntaxin 4 lacking its N-terminal domain is unable to be diverted into this pathway. Consequently, mutant syntaxin 4 would be expected to accumulate in the TGN and – possibly by an overflow mechanism – reach the surface in a non-polarized fashion using non-specific trafficking pathways. Since the intracellular co-localization between furin and syntaxin 4-Δ29 is not complete we cannot exclude the possibility that the retaining compartment is, at least in part, the recycling compartment.

This view is consistent with the recent findings by Torres et al. [29]. These investigators also found that several syntaxin 4 mutants that lack binding ability to munc18c are significantly retained intracellularly. However, they also found that the ability to interact with Munc18c does not strictly correlate with the correct basolateral sorting of syntaxin 4 mutants. Altogether, these results suggest a model in which the binding to Munc18c is required for sorting of syntaxin 4 into basolateral transport carriers in the TGN or post-TGN compartments during the initial targeting of newly synthesized syntaxin 4. Lack of Munc18c binding will prevent the efficient exit from this compartment leading to intracellular accumulation. However, Munc18c-binding is not required for subsequent basolateral sorting of syntaxin 4 mutants that have escaped the TGN block. Such sorting may occur in endocytic and recycling pathways.

This model is further supported by the previous finding that expression of syntaxin 1A in cells that lack its normal Munc18a binding partner results in intracellular retention of syntaxin 1A in the TGN [38]. In contrast, we have previously shown that apical targeting of syntaxin 3 is independent of binding to Munc18b [11]. It is therefore likely that syntaxin/SM interactions are required for intracellular trafficking of some but not all syntaxin/SM pairs.

LLC-PK cells lack expression of the μ1B subunit of AP1B and mistarget AP1B-dependent basolateral proteins [20]. AP1B is involved in biosynthetic and post-endocytic sorting of basolaterally targeted proteins [12], [39] with tyrosine motifs (low-density lipoprotein receptor (LDLR)) and non-tyrosine motifs (transferrin receptor (TfR). We found that the localization of syntaxin 4 is nonpolarized in LLC-PK cells and that basolateral-specific sorting can be restored by re-expression of μ1B. This indicates that basolateral sorting of syntaxin 4 is AP1B-dependent. We investigated the possibility of a protein-protein interaction between AP1B and syntaxin 4 but, under the conditions chosen, failed to reproducibly detect a stable complex (data not shown, unpublished data). Even though we cannot exclude a direct interaction between syntaxin 4 and AP1B, it is possible that this interaction is indirect.

Disruption of basolateral-specific targeting of syntaxin 4 (using the syntaxin 4-Δ29 deletion mutant) led to the inability of MDCK cells to form a polarized morphology in 3D cyst culture, and to a delay in tight-junction formation in 2D culture. This suggests that the restriction of syntaxin 4 to the basolateral domain is a requirement for the establishment of epithelial polarity. Recent results by Torres et al. are consistent with this interpretation as these investigators found that knock-down of syntaxin 4 leads to aberrant, intracellular localization of the tight junction proteins occluding and claudin 2 [29]. This suggests that targeting of these tight junction proteins is dependent on syntaxin 4, and that the ability to polarize is perturbed if syntaxin 4 expression or polarity is disrupted. Previously, we reported similar disruptions of epithelial polarity when the apical SNARE syntaxin 3 is mis-targeted [11]. Altogether, this suggests that syntaxins 3 and 4 are a pair of polarity proteins whose proper intracellular trafficking is intimately involved with the formation of a polarized epithelial phenotype.

When mutant syntaxin 4-Δ29 is expressed in non-polarized or early polarized MDCK cells (1–2 days after plating), the cells fail to polarize (data observed, unpublished data). However, when Syntaxin 4-Δ29 is expressed at a later time point, after polarity is well established (4 days after plating), the cells will maintain their morphology (Fig. 5, 6). This suggests that basolateral-specific targeting of syntaxin 4 is particularly important during early stages in the establishment of epithelial polarity.

Altogether, the results presented here elucidate the mechanism of basolateral targeting of syntaxin 4, its dependence on AP1B and direct trafficking to the basolateral membrane in the biosynthetic pathway which likely involves sorting in the TGN.

## Materials and Methods

### Reagents and antibodies

9E10 anti-myc monoclonal antibody (for immunoprecipitations and western-blotting), R40.76 anti-ZO1 rat antibody and the 3P21D8 anti-gp135 monoclonal antibody were obtained from the American Type Culture Collection (Manassas, VA). Monoclonal antibody anti-Myc tag, clone 4A6 from Millipore was used for immunofluorescence. Affinity-purified polyclonal antibody against a C-terminal peptide of human SNAP-23 has been described previously [40]. The polyclonal anti-Furin Convertase antibody was purchased from Thermo Scientific, IL. Polyclonal antibody against Munc18-3 (Munc18c) was a kind gift from Dr. Ulrich Blank (INSERM U699, Faculté de Médecine Paris 7). Secondary antibodies conjugated to DyLight 488 or 594 and peroxidase were from Thermo Scientific and Jackson ImmunoResearch Laboratories, respectively. Collagenase type VII, protease inhibitors, doxycycline and nitrocellulose membranes were obtained from Sigma-Aldrich.

### MDCK and LLC-PK cell culture and transfection

MDCK clone #11 cells were cultured in minimal essential medium (MEM) (Cellgro, Mediatech, Inc., Manassas, VA) containing 5% fetal bovine serum (FBS) (Omega Scientific Inc., Tarzana, CA), penicillin and streptomycin (Cellgro, Mediatech) at 37°C and 5% CO_2_. Doxycycline-inducible stable cell lines for syntaxin 4-WT and syntaxin 4-Δ29 were made as described previously [11]. For transient transfections, cells were seeded on Transwell filters (12 mm diameter, 0.4 µM pore size; Costar Corning) and immediately mixed with the transfection agent Exgen500 (Fermentas, Hanover, MD) and plasmid DNA in 500 µl of media containing 15% FBS. Fresh media with or without doxycycline was added after six hours of transfection. The cells were cultured for a total of 30 hrs until they were polarized. All transient transfection experiments were repeated at least three times.

LLC-PK1 cells stably expressing either μ1A or μ1B have previously been described [20]. These cells were further stably transfected for the expression of epitope-tagged syntaxin 3 and syntaxin 4 (more information about these plasmids in Low et al. [10]). Cells were maintained in MEM containing 5% FBS, penicillin and streptomycin at 37°C and 5% CO_2_. For localization studies of these SNARE proteins, cells were grown on Transwell filters, fixed, permeabilized and immunostained as indicated in the immunofluorescence microscopy section.

### Mutagenesis

All expression constructs are based on human syntaxin 4 (U07158, mRNA) using a pcDNA4-TO expression vector (Invitrogen) that was modified for the addition of two C-terminal myc epitope tags and one hexa-histidine tag. Deletion mutants were made by PCR as per standard procedures. Briefly, primers were designed with complementary sequences and restriction enzyme sites at ends. PCR reactions were performed using Pfu polymerase (Stratagene, La Jolla, CA) for 20–25 cycles. Product and vector plasmid were digested using desired restriction enzymes and then ligated with T4 DNA ligase. All constructs were confirmed by sequencing.

### Surface delivery assay

An assay to quantify the kinetics of surface delivery of newly synthesized syntaxin 4 was established by modification of a protocol for measuring surface delivery of the polymeric immunoglobulin receptor in MDCK cells [40]. Briefly, MDCK cells stably expressing myc-tagged syntaxin 4 were cultured on Transwell filters for 72 hours. After 12 hours of induction with doxycycline for the expression of syntaxin 4, cells were starved for 30 min in methionine-deficient media (DMEM Gibco/Invitrogen Corporation N.Y.). After starvation, cells were metabolically labeled for 15 minutes with [^35^S]-methionine (Amersham Biosciences) followed by a chase with unlabeled methionine for different time intervals. 9E10 anti-myc antibody was present throughout the chase in either the apical or basolateral media compartment. Antibody binding was allowed to proceed for 60 minutes on ice and then excess antibody was washed away. Cells were lysed in a buffer containing Triton X-100 with the addition of MDCK cell lysates containing an excess of unlabeled myc-tagged syntaxin. Antibody-tagged syntaxin molecules that had been exposed to the surface were precipitated with Protein A-Sepharose. The remaining syntaxin molecules that had not reached the surface were subsequently immunoprecipitated with fresh antibody and Protein G-Sepharose. Immunoprecipitates were separated by SDS-PAGE, gels were dried and radioactive bands were imaged using a Molecular Imager FX (Bio-Rad Laboratories). Images were quantitatively analyzed using Quantity One analyzing software (Bio-Rad Laboratories).

### Immunofluorescence microscopy

For surface staining, MDCK cells on Transwell filters were incubated on ice for 1 hr with the anti-myc epitope antibody 9E10 diluted in MEM containing 20 mM Hepes and 0.6% BSA with gentle shaking. The cells were washed with MEM four times for 10 minutes. Afterwards, the cells were fixed with 4% paraformaldehyde (Sigma-Aldrich) at 4°C for 25 minutes. After quenching in PBS containing 75 mM ammonium chloride and 25 mM glycine, cells were blocked and permeabilized with PBS containing 3% BSA and 0.2%Triton X-100. Filters were cut out and incubated overnight with primary antibodies in blocking solution at 4°C. Then filters were washed with washing solution (PBS, 0.05% Triton X-100 and 0.7% fish skin gelatin) four times during 5 minutes followed by incubation with fluorescent-labeled secondary antibodies plus DAPI 0.2 µg/ml at 37°C for 1 h. After washing, membranes were post-fixed 5 minutes with 4% paraformaldehyde and mounted in coverslips using ProLong Gold antifade reagent (Molecular Probes). For intracellular staining, stable cell lines were culture on coverslips for at least 4 days and then expression of the protein was induced for 8 hours with doxycycline. After induction, cells were fixed and labeled as described above.

Images were acquired either with a Lecia-TCS-SP2 confocal microscope (Leica Microsystems Heidelberg GmbH) or an Olympus IX81, equipped with Disk Spinning Unit (Olympus, USA) microscope at room temperature. Projection images were constructed using either Leica confocal software or IPLab software (BD Biosciences, MD, USA). Using Adobe Photoshop software, histograms were linearly adjusted for optimal representation of the 8 bit signals. Individual channels were overlaid in RGB images, and composites of panels were made for final figures.

### Immunoprecipitation

For immunoprecipitation assays polarized stable cell lines were lysed for 30 min under rotation at 4°C in lysis buffer containing 50 mM Hepes-KOH pH 7.4, 50 mM potassium acetate, 1% Triton X-100. This buffer was supplemented with a protease inhibitor cocktail and PMSF. The lysate was centrifuged for 10 minutes at 10,000 g and the supernatant was pre-cleared by a 20 min incubation with CL-2B beads (GE Healthcare). Then, the lysate was incubated overnight at 4°C with anti-myc antibody cross-linked to protein A-Sepharose beads (GE Healthcare). Subsequently, the beads were washed three times with lysis buffer containing Triton X-100 and one last time with lysis buffer without Triton X-100. Immunoprecipitated complexes were analyzed by SDS-PAGE and western blotting for myc, SNAP-23 and Munc18c proteins.

### Transepithelial electrical resistance (TEER) measurements

Cells were grown on Transwell filters (12 mm diameter, 0.4 mM pore size) for at least 72 hrs, followed by induction with doxycycline for 8 hrs. Cultures were switched to S-MEM low-calcium medium (Cellgro, Mediatech) for 15 hrs, resulting in the loss of tight junctions. After this incubation cultures were switched back to normal calcium and the reestablishment of tight junctions was monitored. TEER was measured in Ω, at 37°C, using an EVOM epithelial voltohmmeter (World Precision Instruments, Inc).

### Cyst culture

For culture in 3D-collagen gels, MDCK cells were seeded at 0.5×10^4^ to 1×10^4^ cells/ml in 80% collagen Type I:PureCol (Inamed) and 20% MEM containing 0.02 M HEPES (pH 7.4) and 0.02 M NaHCO_3_ on 48 well plates (Costar, Corning Incorporated, NY). The cultures were kept at 37°C for 30 minutes to solidify the collagen, and then media containing MEM (with 5% FBS and penicillin and streptomycin) was added. Two days after seeding, gene expression was induced by adding doxycycline and the cultures were continued for a total of 7–10 days. For immunostaining of MDCK cells in 3D-collagen cultures, the collagen was digested with 10 U/ml of collagenase type VII (Sigma-Aldrich, USA) for 10 minutes. After digestion, gels were fixed with 4% paraformaldehyde (Sigma) for 30 min. Immunostaining was done with extended primary and secondary antibody incubation times and washing (24 hr incubation for antibodies and 4×washing for 30 min). Gels were mounted using ProLong Gold antifade reagent (Molecular Probes).
